# Organic Acid-Based Anodization Process to Produce Bioactive Oxides on Titanium Implants

**DOI:** 10.3390/ma18225190

**Published:** 2025-11-15

**Authors:** Arunendu Ettuthaiyil Sambasivan, Amisha Parekh, Amol V. Janorkar, Michael D. Roach

**Affiliations:** Department of Biomedical Materials Science, University of Mississippi Medical Center, Jackson, MS 39216, USA

**Keywords:** carbonate-substituted apatite, tricalcium phosphate, Ca-incorporation, Mg-incorporation, organic acid-based anodization, biocompatibility

## Abstract

Titanium implants are widely used in medicine because of their favorable mechanical properties and biocompatibility; however, the rapidly forming titanium oxide coatings do not provide an ideal bioactive surface to stimulate osseointegration. This study aims to enhance titanium implant osseointegration through anodization processes designed to incorporate elements and compounds present within human bone into the surface oxides. Commercially pure titanium grade 4 (CPTi) discs were anodized in either oxalic, malic, or ascorbic acid-based electrolytes. Each resulting oxide exhibited complex surface topographies. EDS analyses revealed that Ca, P, and Mg bone chemistry dopant elements were incorporated into each of the oxide coatings. X-ray diffraction analyses revealed combinations of anatase and calcium titanate compounds present in each oxide. Additionally, two of the anodized oxides showed calcium oxide formation, and one oxide also revealed tricalcium phosphate (α-TCP) and hydroxyapatite (HA) formation. Subsequent FTIR spectroscopy analyses revealed carbonate substitution peaks to be present in two of the oxides. This finding indicated that the TCP and HA compounds shown in the XRD analyses of one oxide represented the formation of bone-like carbonated calcium phosphate compounds. A 21-day cell culture study showed favorable cell culture responses for each of the organic-acid-based anodized oxides. Moreover, two of the oxides showed good cytocompatibility and early osteogenic differentiation compared to non-anodized titanium controls. Thus, the organic acid anodization processes developed in this study show promise to enhance future titanium implant clinical outcomes.

## 1. Introduction

Implants are surgically inserted into the body to replace, support, or enhance damaged or missing tissues or organs. Implant success strongly depends on developments at the implant-bone interface. In many cases, successful implantation can be defined by osseointegration [1,2]. Inadequate osseointegration may lead to localized inflammation or fibrous capsule formation that results in early implant failures [1,3,4]. Most dental implant failures have been reported to occur in the early healing phase before prosthetic placement [5]. A 2023 USA retrospective study compiling data on over 50,000 dental implants revealed a 1.40% failure rate [6]. A comparable failure rate was also shown in a 2025 Israeli study of dental implants, revealing a 2.21% overall failure rate, with 1.56% failures occurring during the osseointegration phase [5].

Titanium alloys are commonly used because of their high mechanical strength, corrosion resistance, strength-to-weight ratio, and biocompatibility [1,7,8]. When exposed to an oxygenated environment such as ambient air, titanium forms a stable amorphous oxide that provides excellent corrosion resistance. However, the naturally forming amorphous oxide is not bioactive and thus does not promote osseointegration [9,10,11].

Incorporation of bone-like chemistries, such as additions of calcium, phosphorus, and magnesium, into titanium surface oxides has shown improved osseointegration [12,13,14,15,16]. Incorporated calcium phosphate-based compounds, which closely resemble the mineral component of bone, have shown properties of osteoconductivity, biocompatibility, and ability to support early-stage bone growth [12,17,18]. Tricalcium phosphate (TCP) and hydroxyapatite (HA) are widely used in bone cements and orthopedic grafts because of their known biocompatibility, beneficial mechanical integration properties, and bio-resorption abilities [17,18]. TCP is naturally found in bone and is an osteoconductive compound [18]. HA coatings on a titanium implant surface have been shown to greatly increase their bioactivity and bone-implant-contact area [19]. An electrophoretically deposited calcium carbonate and nanohydroxyapatite-containing coating on titanium implants in rabbit femurs showed earlier cellular maturation and mineralization compared to non-coated implant counterparts [20]. Commonly used HA coating processes for titanium implants include dip coating, pulsed laser deposition, electron beam deposition, and plasma spraying [1,19,21,22]. Most of these techniques have the disadvantages of requiring expensive, specialized equipment and extensive specimen preparation protocols. Many of these HA coating processes also suffer from low-adhesion strengths to titanium implant substrate materials. The less-than-desirable coating adhesion strength of these HA coatings can lead to coating delamination from the implant substrate and subsequent early failure [23,24].

Anodization processes provide an alternative single-step coating approach to modify the surface topography, surface chemistry, and surface oxide crystallinity on titanium implant materials [25,26]. Anodization has been shown to produce complex multileveled micro and nano-scale titanium oxide surface topographies, which enhanced mechanical bone interlocking, improved protein absorption, and increased osteoblast cell attachment and differentiation [27,28]. Both single-step anodization processes and anodization processes with subsequent post-treatments have shown the ability to incorporate Ca, P, and Mg bone chemistry dopant elements into the oxide layers [29,30,31]. Some anodization processes have incorporated HA directly into the oxides [12,17,18,32,33,34,35,36,37,38,39,40,41]. Recent anodization studies that have incorporated HA into the oxides have shown an increased HA interfacial bonding strength compared to conventional plasma spray HA coating methods [31,40].

In previous studies, we used citrus fruit-based juice electrolytes and citric acid-based electrolytes to produce anodization coatings containing beneficial calcium phosphate compounds, including tricalcium phosphate or hydroxyapatite, on titanium substrates [42,43]. Moreover, magnesium dopants were incorporated into the calcium phosphate-containing oxides [33]. Other organic acids, inclusive of oxalic acid, ascorbic acid, and malic acid, are also known for their involvement in biological processes [42,43,44,45]. Oxalic acid is commonly found in fungal secretome and contributes to the processes of biomineralization and bio-weathering [42]. In our previous anodization study, which incorporated oxalic acid as an electrolyte component, the resulting phosphorus-doped oxides exhibited enhanced osteoblast mineralization compared to non-anodized titanium substrates [31]. Ascorbic acid, vitamin C, plays a critical role in collagen synthesis and is a vital component in connective tissues and bones. Ascorbic acid also influences osteogenic cell differentiation [43,44]. Malic acid is a natural component of the citric acid cycle and possesses antioxidant and antimicrobial properties [45]. The primary purpose of the present research was to compare the oxide surface characteristics and cellular responses of oxides formed in anodization processes using oxalic, ascorbic, and malic acid-based electrolytes. A secondary objective was to compare these organic acid-based anodization coatings to those formed in our previous studies using citrus fruit-based and citric acid-based electrolytes. It was hypothesized that the anodization processes in oxalic, ascorbic, and malic acid-based electrolytes would each form oxide coatings containing tricalcium phosphate and hydroxyapatite compounds that would exhibit enhanced cellular responses compared to non-anodized titanium substrates.

## 2. Materials and Methods

### 2.1. Titanium Specimen Preparation

In total, 3 mm thick discs were sectioned from an 11 mm diameter commercially pure titanium grade 4 (CPTi) bar stock. Any rough edges generated during sectioning were ground down using 120-grit SiC paper. Ground discs were ultrasonically cleaned with a laboratory detergent solution (Alconox^®^, White Plains, NY, USA), rinsed in distilled water, and blown dry using laboratory air. Cleaned discs were then pre-activated for anodization using a nitric acid–hydrofluoric acid (10:1 ratio) solution (TURCO NITRADD, Henkel Corporation, Madison Heights, MI, USA) 30 s dip prior to electrolyte immersion.

### 2.2. Anodization

Anodization processes utilized in this study were performed in a reaction cell made up of an activated CPTi disc anode and two CPTi strip-shaped cathodes (https://www.onlinemetals.com, accessed on 29 October 2025, Ackworth, GA, USA). Anodization processes were applied with a DC rectifier (350 V, 10 A, Dynatronix, Amery, WI, USA). For each anodization process, pulsed galvanostatic waveforms at a 700 mA/cm^2^ current density were applied over a 28% duty cycle and a 7.2 Hz frequency for 120 s. Multiple anodized disc specimens (n = 3) were produced for each group. The electrolytes used in each anodization process are compiled in Table 1. Each electrolyte was a mixture of three components including an organic acid, either oxalic acid (Oxalic acid dihydrate, Alfa Aesar, Ward Hill, MA, USA), ascorbic acid (99+%, Thermo Fisher Scientific, Ward Hill, MA, USA) or malic acid (99+%, Thermo Fisher Scientific, Fair Lawn, NJ, USA), magnesium phosphate dibasic trihydrate (96.0%, Spectrum Chemical, New Brunswick, NJ, USA) and calcium acetate (99% Spectrum Chemical, New Brunswick, NJ, USA). The electrolyte components were mixed together in 500 mL of distilled water (Table 1). The molarities listed in Table 1 are representative of the final molarities within the 500 mL electrolyte.

### 2.3. Anodized Oxide Surface Characterization

Glancing-angle X-ray diffraction (XRD; XDS 2000, Scintag, Franklin, MA, USA) was used to verify the crystalline phases present in each anodized oxide (n = 3). Each anodized specimen was rotated by 1° relative to the incident Cu-Kα X-ray beam (λ = 0.154 nm) to optimize the interaction depth and diffraction signal to the oxide coatings. XRD scans were performed across a 2θ range of 20° to 60° to capture diffraction peaks relevant to crystalline titanium oxide, calcium compounds, and calcium phosphate compounds. Oxide surface topographies were examined with scanning electron microscopy (SEM; Zeiss Supra 40, Jena, Germany) and laser confocal microscopy (Keyence VK-X3000, Osaka, Japan). For macroscopic imaging of the oxide surfaces, laser confocal microscopy was used to provide the general surface appearance of the anodized disc specimens. For high-magnification imaging of multileveled micro- and nano-scaled oxide surface features, anodized disc specimens were first sputter-coated with 4 nm gold to minimize surface charging effects in the high-vacuum SEM chamber environment. It should also be noted here that these gold-coated SEM specimens were not used for the macroscopic laser confocal imaging analyses or the subsequent surface roughness, surface chemistry, or molecular structure analyses of the anodized oxides. The oxide surface roughness characteristics were measured in triplicate areas of each group using laser confocal microscopy (LCM; Keyence VK-X3000, Osaka, Japan). The oxide surface chemical compositions were verified using energy-dispersive X-ray spectroscopy (EDS; EDAX APEX Software Suite, Advanced Version 2.5.1001.0001, Mahwah, NJ, USA). Average surface concentrations of key elements, as well as the average Ca/P ratios, were compiled for each test group. Attenuated total reflectance Fourier transform infrared spectroscopy (ATR-FTIR; Spectrum 100, PerkinElmer, Waltham, MA, USA) was utilized to assess the molecular structures in each group. ATR-FTIR spectra were collected over a range of 650–4000 cm^−1^ using a 4 cm^−1^ resolution.

### 2.4. Anodized Oxide Thickness Evaluation

Anodized oxide specimens from each group were cross-sectioned, ground with 120-grit SiC paper, and mounted in conductive epoxy (Polyfast, Struers, Cleveland, OH, USA). The mounted anodized specimen cross-sections were then rotary polished to a 0.02 µm finish in a colloidal silica suspension (Struers, Cleveland, OH, USA). Eight cross-section images capturing views of each anodized oxide group were acquired in the SEM. Five oxide thickness measurements were completed on each image from each group, yielding a total of 40 thickness measurements per oxide group (n = 40).

### 2.5. Anodized Oxide Adhesion Quality Assessment

The quality of oxide adhesion for each group was assessed using a Rockwell C indentation methodology in accordance with the VDI 3198 standard [46,47,48,49]. This indentation-based technique evaluates the qualitative adhesion strength of each oxide by driving a Rockwell C hardness indenter under a 150 kg load through the coating and into the titanium substrate. After testing, the surface damage present around the surface indentation is qualitatively examined under an optical microscope, and the observed damage levels are compared with VDI 3198 reference charts [49]. This VDI coating adhesion test methodology has been routinely used to evaluate other anodization coatings [46,47,48].

### 2.6. Osteoblast Culture

Pre-osteoblastic mouse cells (MC3T3-E1, Subclone 4; American Type Culture Collection, Manassas, VA, USA) were grown to confluency in alpha-modified Eagle’s minimum essential medium, enriched with 10% fetal bovine serum (Gibco, Life Technologies Corporation, Grand Island, NY, USA) and 1% penicillin–streptomycin (MP Biomedicals, Fountain Parkway, Solon, OH, USA), which is known as the maintenance media. Cultures were incubated in a humidified atmosphere containing 5% CO_2_ at 37 °C. Representative anodized specimens from each group and non-anodized CPTi control specimens were sonicated for 5 min in distilled water and sterilized via autoclaving for 15 min at 121 °C (Pelton & Crane) prior to cell seeding. Anodized and control specimens were seeded with pre-osteoblasts at a density of 50,000 cells/cm^2^. Three independent disc specimens (n = 3) from each anodized oxide or non-anodized CPTi control group were analyzed for the MTT and biochemical analysis assays. Triplicate technical replicates were measured for each of the disc specimens (n = 3) representing each test group, resulting in nine technical replicate values (n = 9). The nine technical replicate values representing each test group were then used for further calculations and statistical analyses. The culture medium used for the MTT assay was the maintenance media. For the other biochemical analyses, the culture medium was the differentiation media, which is maintenance media supplemented with ascorbic acid (L-Ascorbic acid, Fisher Scientific, Fair Lawn, NJ, USA) and β-glycerophosphate (SIGMA-ALDRICH Co, St. Louis, MO, USA). The cell culture medium for the MTT assay and the biochemical analyses was replaced every 48 h for the duration of the 21-day study.

### 2.7. Cell Viability Testing

Quantitative assessment of cell viability was performed on day 7 and day 21, via the CyQUANT™ MTT Cell Viability Assay Kit (Invitrogen, Thermo Fischer Scientific, Life Technologies Corporation, Eugene, OR, USA), following established protocols [50]. After removing the culture medium, MTT solution was pipetted into each well in a cell culture plate, and the anodized and control specimens were incubated at 37 °C for 4 h in 5% CO_2_. Media and MTT solution-only wells, with no anodized or control disc specimens, were also subjected to the 21-day study to provide additional controls. After the incubation period, the MTT solution was pipetted out, and dimethyl sulfoxide (DMSO; Sigma Aldrich, St. Louis, MO, USA) was introduced and incubated for 10 min to dissolve formazan crystals. The resulting solutions were then centrifuged to collect the supernatant. Three 100 µL technical replicates representative of each disc specimen from each oxide group, non-anodized CPTi control group, or media only control group were then transferred to a 96-well plate (n = 3). An ELx800 plate reader (Biotek, Winooski, VT, USA) was utilized to record absorbance values at 540 nm for the nine technical replicate values representing each group (n = 9).

### 2.8. Biochemical Analyses

DNA content was measured at 7, 14, and 21 days using the CyQUANT™ Cell Proliferation Assay Kit (Invitrogen). At every time point, cell-seeded specimens were rinsed with PBS, detached with trypsin–EDTA (Gibco, Life Technologies Corporation, Grand Island, NY, USA), neutralized with FBS-supplemented DMEM (Gibco), centrifuged, resuspended in PBS, and lysed by 30 s of sonication at 10% amplitude. Cell-seeded specimens were then incubated with CyQUANT GR dye (Invitrogen). The fluorescence values for three technical replicates from each specimen (n = 3) of each group, resulting in nine technical replicates (n = 9) per group, were then quantified with the FLX-800 plate reader (Biotek) at 485 nm excitation/520 nm emission to obtain the DNA content for each group. ALP activity was determined on cell culture days 7, 14, and 21 using the QuantiChrom kit (BioAssay Systems, Hayward, CA, USA). Cell lysates were incubated with p-nitrophenyl phosphate (QuantiChrom kit) substrate in assay buffer. The absorbance values for three technical replicates representative of each specimen (n = 3) of each group were then measured at 405 nm using the ELx800 plate reader after 0 and 4 min (n = 9). The ALP activity values were calculated per the manufacturer’s protocol and normalized with the average DNA content values for each group.

### 2.9. Statistical Analyses

One-way ANOVA (α = 0.05) with post hoc Tukey analysis was used to verify significant differences in oxide surface roughness values, EDS-derived surface Ca/P dopant uptake ratios, and oxide coating thickness values for each of the groups in the present study. A two-way repeated measures ANOVA (α = 0.05) with post hoc Bonferroni analysis was utilized to assess the influence of the specimen group and day of cell culture on the MTT, DNA, and ALP assay results, since unequal variance values were shown for the different anodized and control specimen groups.

## 3. Results and Discussion

### 3.1. Anodized Oxide Characterization

#### 3.1.1. Oxide Structural Analysis

**Figure 1:**
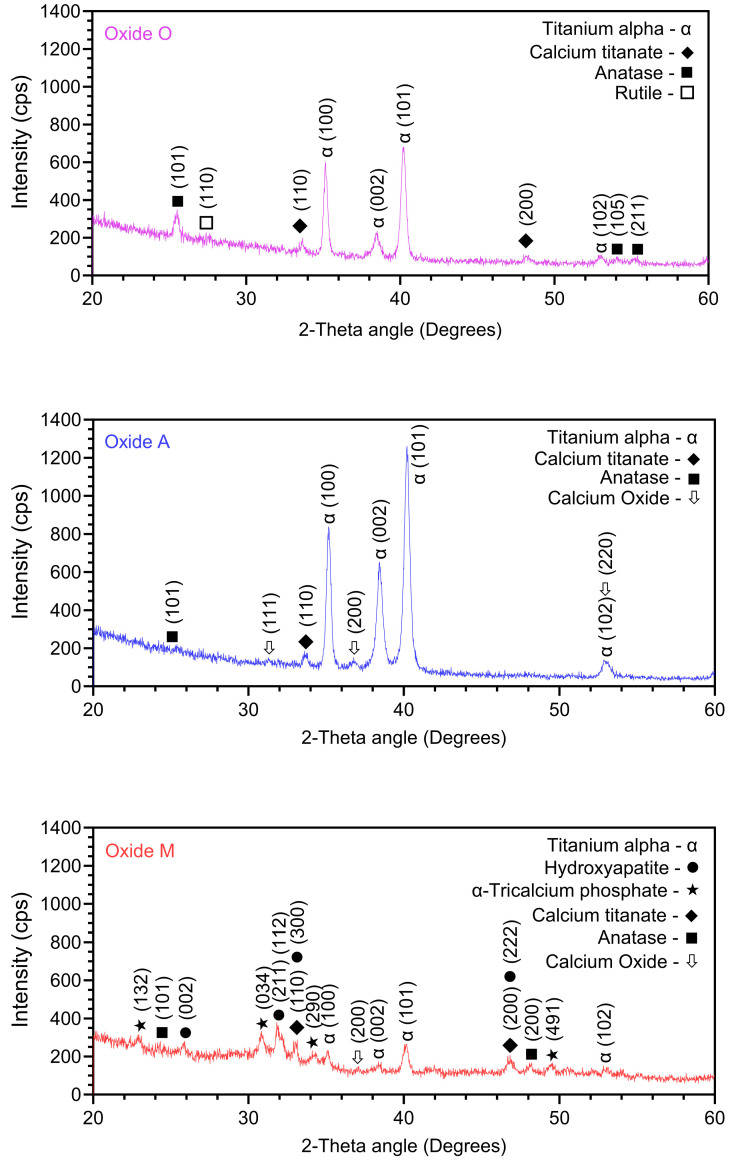
XRD spectra representative of each oxide. Anatase, calcium titanate, and α-titanium phase peaks were shown in each oxide. Additionally, Oxide O also showed rutile peaks, Oxide A showed Ca-O peaks, and Oxide M showed Ca-O, α-TCP, and HA peaks.

XRD oxide crystallinity results are shown in Figure 1. α-titanium phase peaks were present in the XRD spectra from each oxide group. However, the relative intensity shown for the titanium peaks in the Oxide M spectra was substantially reduced, indicating that a thicker oxide was covering the CPTi substrate. Small peaks indicative of anatase and calcium titanate formation were also present in each oxide. Oxide O also revealed a small amount of rutile to be present. Deposited anatase-phase thin films have been shown to promote better osteoblast compatibility than rutile-phase films in terms of cellular adhesion, proliferation, differentiation, mineralization, and osteogenesis-related gene expression in cellular assays [51,52]. Hydrothermally deposited calcium titanate-containing coatings on 3D printed TAV substrates have been shown to improve osteogenic differentiation in pre-osteoblast cells compared to non-coated controls in a rat femoral study [53]. Finally, enhanced bone ingrowth, neurovascular integration, and osseointegration have also been shown for calcium-titanate coatings [53].

Oxide A and Oxide M exhibited the formation of calcium oxide (Ca-O) peaks (Figure 1). Nano-scaled thickness Ca-O films on titanium substrates were shown to increase osteogenic differentiation of bone marrow stem cells and localized bone integration compared with non-coated titanium controls [54]. Oxide M also revealed the formation of α-Tricalcium phosphate (α-TCP) peaks and HA peaks. Thus, the Oxide M anodization coatings revealed the same calcium phosphate compounds formed within the oxides as the previous oxides formed in our citrus fruit-based and citric acid-based electrolyte anodization studies [31,41]. However, the intensities shown in the Oxide M α-TCP and HA XRD peaks were reduced in comparison to those shown for citrus fruit-based and citric acid-based oxides. α-TCP has shown the ability to improve bone cell recruitment, adhesion, and differentiation. HA-coated implants have shown enhanced osteoblast viability, differentiation, and bone mineralization [55]. In vivo studies in rabbit and rat models exhibited better bone-to-implant contact (BIC) for HA-coated titanium implants when compared with non-coated titanium controls. [20,56,57]. Since the Oxide M group in the present study was shown to exhibit these beneficial bioactive compounds, it shows much promise to promote healing in future implant applications.

#### 3.1.2. Anodized Oxide Surface Topographies

**Figure 2:**
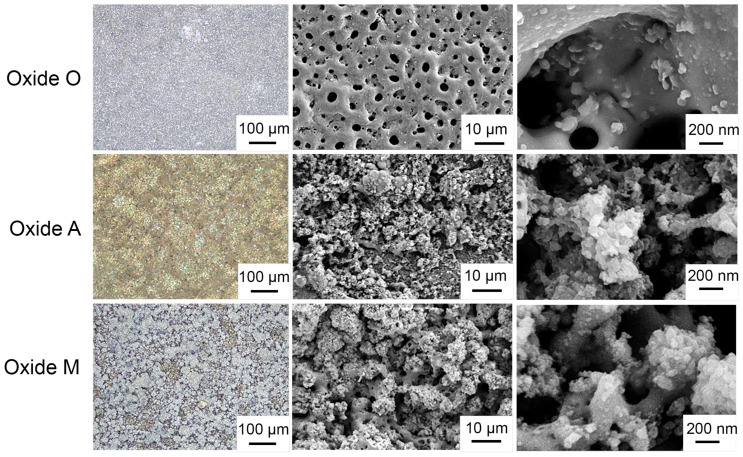
Representative laser confocal microscopy and SEM images for each anodized oxide. The left column shows macro-scaled laser confocal microscope optical images of each oxide. The middle and right columns show micro- and nanoscale SEM images of each oxide. Oxide O revealed a micro-scaled porosity nested with nano-scaled surface features., Oxides A and M revealed small, uniformly distributed, micro- and nano-scaled cauliflower-shaped deposits across their surfaces.

Representative laser confocal microscope and SEM images for each group are provided in Figure 2. The laser confocal macroscopic images in the left column showed a greyish-white surface visual appearance for Oxide O and Oxide M. In contrast, Oxide A revealed a greyish-golden appearance indicative of the presence of a thinner gold-colored anodized oxide coating. Note again that the anodized disc specimens used for the laser confocal macroscopic image analyses were not gold-coated prior to examination. Thus, the oxide A anodization coating was sufficiently thin to provide a macroscopic gold-colored visual appearance for this oxide group. Thus, this finding agreed well with the higher intensity α-titanium XRD peak shown for Oxide A in Figure 1, which was also indicative of a thin oxide coating on the CPTi substrate material.

SEM images in the center and right columns of Figure 2 revealed each oxide group to exhibit complex micro- and nanoscale surface topographies. Oxide O surfaces showed micro-scaled surface porosities nested with nano-scaled surface features. In contrast, Oxides A and M revealed small micro- and nanoscale, uniformly distributed, cauliflower-shaped deposits. Thus, the micro- and nano-scaled surface topographies shown for Oxides A and M were similar to those shown for our citrus fruit-based and citric acid-based oxides in previous studies [31,41]. The nano-scaled surface features shown in Oxide O revealed similar morphologies to the uniformly distributed cauliflower-shaped features on Oxides A and O.

#### 3.1.3. Anodized Oxide Surface Roughness

**Figure 3:**
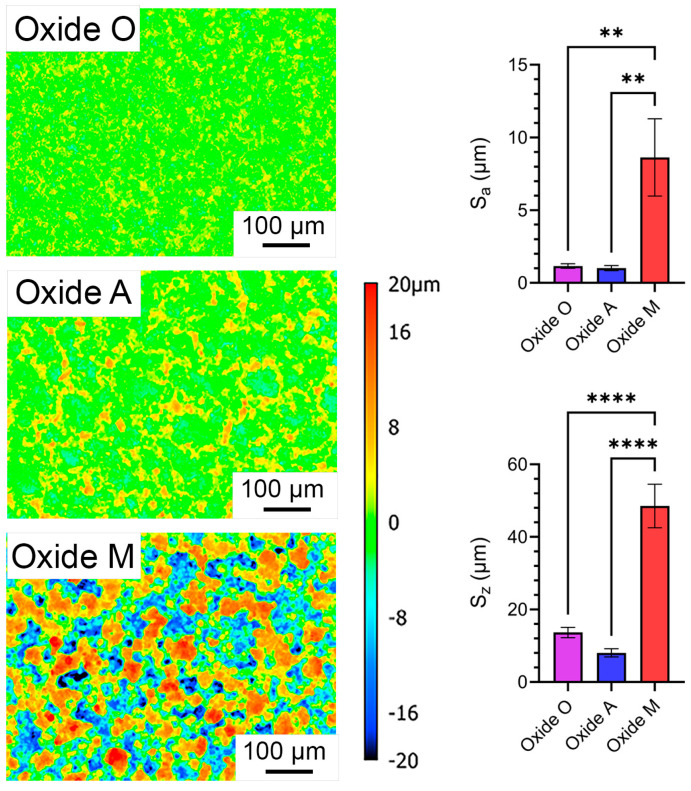
Compiled surface roughness results for each group. The left column provides representative surface height maps and a standardized reference scale. The right column provides the average surface roughness, S_a_, and the peak-to-valley surface roughness, S_z_, comparisons between the oxide groups. ** symbols indicate statistically different groups with a significance level of *p* < 0.01. **** symbols indicate statistically different groups with a significance level of *p* < 0.0001.

A comparison of the surface roughness results for the oxide groups is compiled in Figure 3. Height maps for each oxide group are shown relative to a standardized −20 to 20 μm reference scale in the center of Figure 3. The relative average surface roughness, S_a_, and peak-to-valley roughness, S_z,_ results for each oxide are provided on the right side of Figure 3. Each oxide group revealed average S_a_ values in the range of 1–10 µm, and thus would be classified as micro-scaled surfaces. Previously, micro-scaled topographies have shown enhanced bone–implant contact, strengthened bone-to-implant surface adhesion strengths, and more predictable long-term clinical outcomes compared to macro-scaled (10 µm–1 mm) and nano-scaled (1–100 nm) surface topographies [58]. While all oxide groups exhibited micro-scaled surface topographies, the Oxide M group exhibited significantly higher S_a_ (*p* < 0.01) and S_z_ (*p* < 0.0001) values compared to Oxide O and A groups. The malic acid-based Oxide M coating showed similar S_a_ and S_z_ values to the citric acid-based oxides formed on CPTi in our previous study [31]. In contrast, the ascorbic acid-based Oxide A coating revealed significantly lower surface roughness values even though similar micro and nano-scaled cauliflower-shaped surface topographical features were still present (Figure 2).

#### 3.1.4. Anodized Oxide Surface Composition

**Figure 4:**
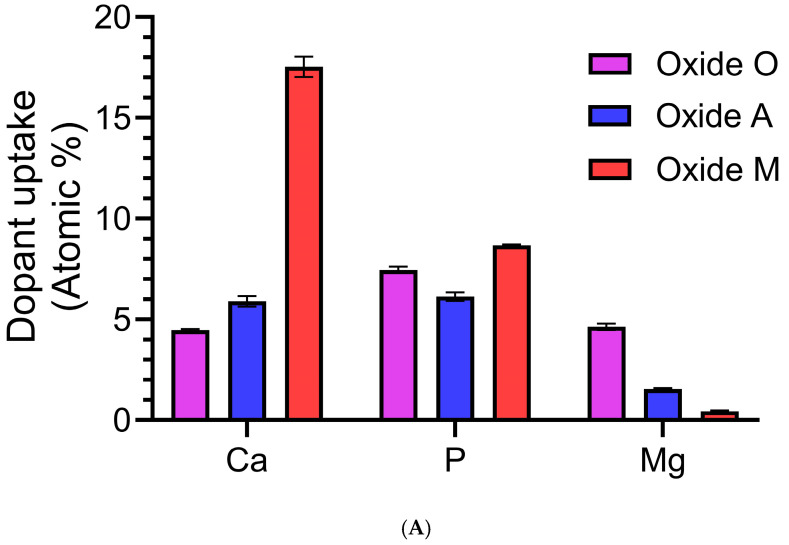

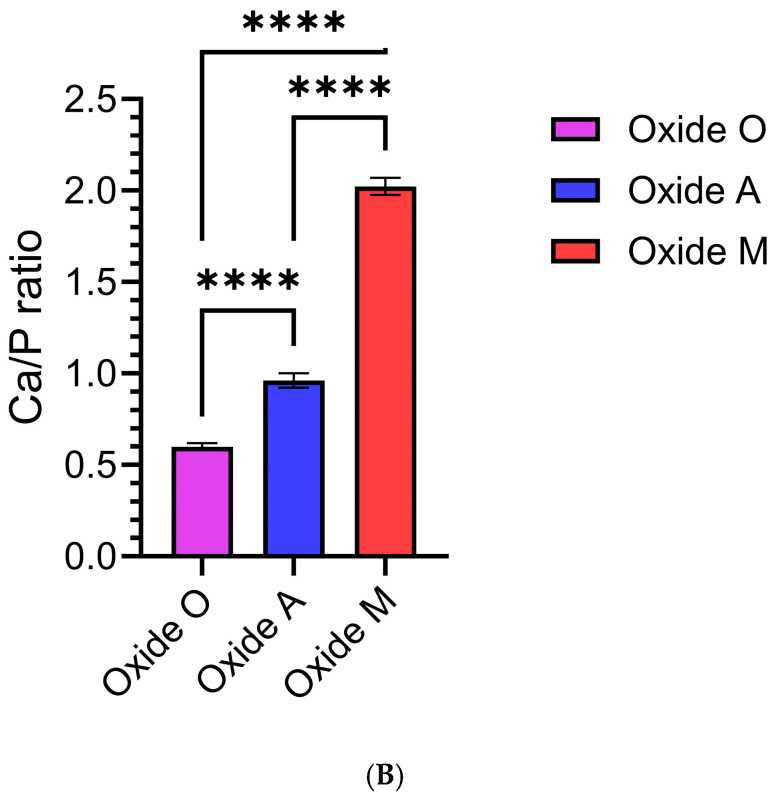
(**A**). Oxide dopant uptake comparison for each oxide group. The amount of Mg dopant uptake was shown to decrease as the uptake of Ca increased, and the levels of P dopant uptake were shown to be relatively similar for each oxide. (**B**). Oxide Ca/P ratio comparison for each oxide. The statistical analysis revealed that each oxide exhibited a significantly different surface Ca/P dopant uptake ratio. The **** symbol represents a significance of *p* < 0.0001.

The EDS surface compositions for each group are listed in Table 2. Comparisons of the relative amounts of Ca, P, and Mg dopant uptake into each oxide, along with the associated surface Ca/P ratios, are compiled in Figure 4A and Figure 4B, respectively. Substantial differences were shown in the amounts of dopant uptake into the oxides produced from the different organic acid-based anodization processes in this study. Oxide O showed Ca, P, and Mg uptake levels of approximately 4, 7, and 5 at %. Ca uptake was shown to be the highest, at a level of approximately 18 at %, in Oxide M, while Mg uptake was shown to be the highest, at a level of approximately 5 at % in Oxide O. The level of P dopant uptake was relatively similar in the oxide surfaces produced in each anodization process, ranging from 6 to 9 at %. The substantial differences shown in the amount of Ca dopant uptake into each of the oxides led to significantly different surface Ca/P ratios in each group. The Ca/P ratios for Oxides O and A were 0.6 ± 0.02 and 1.0 ± 0.04, while the average Ca/P ratio for oxide M was 2.0 ± 0.05. Thus, only Oxide M was shown to exhibit a Ca/P ratio close to the stoichiometric Ca/P ratio of hydroxyapatite (1.67 at %) [59,60]. Another interesting trend that emerged from this analysis was a decreased Mg dopant uptake as the Ca-dopant uptake was shown to increase. Ca, P, and Mg are each important components of the composition of bone and have been shown to regulate osteoblast and osteoclast activity, control matrix mineralization, and enhance expression of certain markers [17,29,61,62]. The lower levels of Ti shown in the surface EDS dataset for Oxide M agreed well with the lower intensity Ti peaks shown in XRD spectra (Figure 1) for the same oxide, and are indicative of a thicker oxide coating. Overall, Oxide O revealed a promising high uptake of the Mg bone mineral, and Oxide M revealed a promising surface Ca/P uptake ratio similar to the ratio commonly found in human bone. Interestingly, the Ca and P dopant uptake into the Oxide M coatings was significantly higher than what was shown in the citric acid-based and citrus-fruit-based oxides on CPTi in our previous studies [31,41]. Nonetheless, the surface Ca/P ratio shown for Oxide M was similar to what was shown for the citric-based and citrus fruit-based oxides [31,41]. It was also interesting to note that the Mg dopant uptake into Oxide O was significantly higher than the Mg uptake into the previous citrus-fruit-based and citric acid-based oxide that used the same concentration of magnesium phosphate as an electrolyte component [31,41]. Thus, the oxalic acid-based anodization process was shown to incorporate substantial Mg into the oxide coatings, but not incorporate the HA or TCP compounds within the surface oxides like the citric and malic acid-based anodization processes.

#### 3.1.5. Anodized Oxide Molecular Structure Analyses

**Figure 5:**
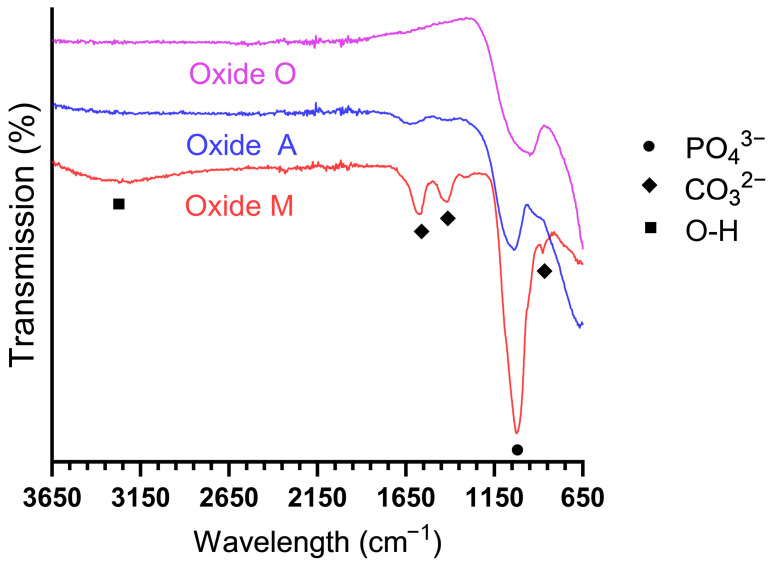
FTIR spectra representative of each oxide. Each oxide exhibited evident phosphate peaks at 1050 cm^−1^. Oxides A and M also showed evidence of carbonate substitutions with peaks at 875, 1450, and 1570 cm^−1^.

Representative FTIR scans are provided in Figure 5. Each oxide revealed prominent phosphate peaks at 1050 cm^−1^, suggesting the incorporation of calcium phosphate compounds within the coatings [37,63]. In Oxide A and Oxide M, carbonate peaks were present at 875, 1450, and 1570 cm^−1^ [37,63,64]. The carbonate substitution peaks were much more prominent in Oxide M compared to Oxide A. Combining this information with the XRD, SEM, and EDS analyses results, it was identified that Ca-O formation in the oxides may represent an intermediate/precursor to the formation of Ca-P-based compounds. Oxide M also revealed a broad and undefined O-H band between 3000 and 3600 cm^−1^, which has been shown to correspond to adsorbed water [37,63,64]. The formation of carbonate peaks, combined with the lack of a hydroxyapatite OH^-^ peak around 3570 cm^−1^, indicated that the HA formed in Oxide M was bone-like carbonated apatite [37,63,64]. The carbonate substitution lowers the crystallinity and improves the solubility and bioactivity of HA [12,65,66]. The Oxide M carbonate peaks shown in the present study were shown to be similar in intensity to those shown for the citric acid-based and citrus fruit-based oxide coatings in our previous studies [31,41]. Bone-like carbonated apatite has been demonstrated to be biocompatible and non-toxic, promoting the adhesion and proliferation of osteoblast cells [66].

### 3.2. Anodized Oxide Coating Thickness

**Figure 6:**
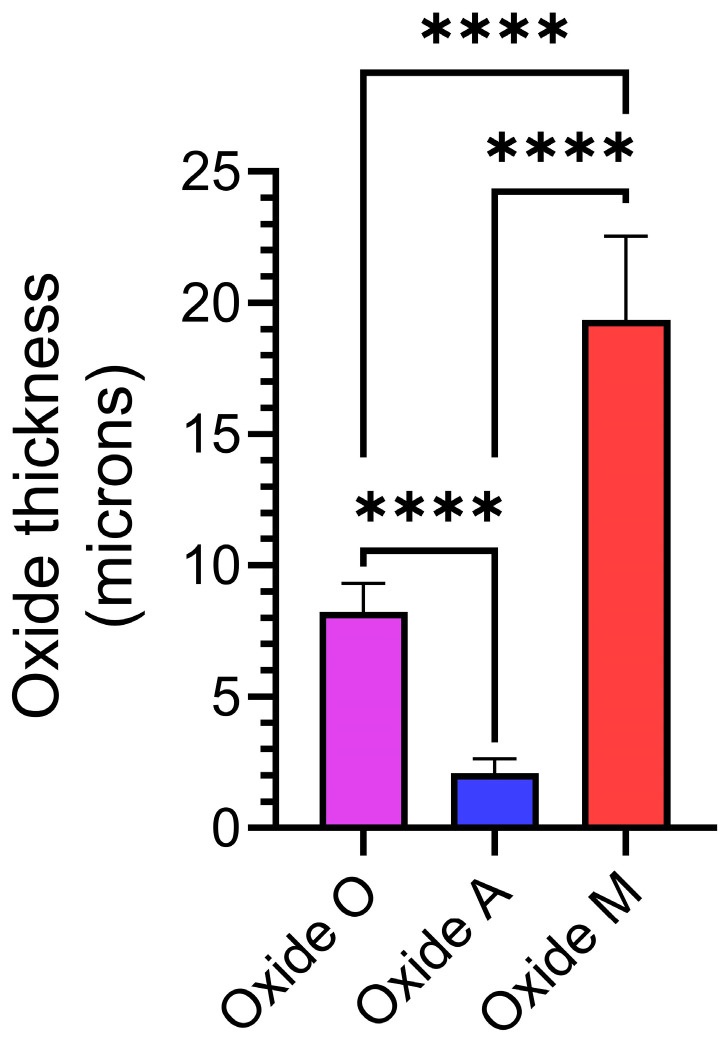
Representative oxide thickness comparisons for each group. The statistical analysis revealed that each oxide group exhibited a significantly different average oxide layer thickness. The **** symbol represents a statistical significance of *p* < 0.0001.

Representative oxide thickness measurements are compiled in Figure 6. Oxide A revealed the thinnest coating (2.098 ± 0.54 µm), which agreed well with the XRD, SEM, and EDS surface results from this oxide. Oxide O showed a significantly higher thickness than Oxide A (*p* < 0.0001). Oxide M revealed the thickest coating (19.37 ± 3.14 µm) in the study, which was significantly thicker than both the Oxide O and Oxide A coatings (*p* < 0.0001).

This finding also agreed well with the previous XRD and surface roughness datasets for Oxide M. It should be noted that even though the oxides produced in each anodization process showed significantly different thickness values, all measured thickness values were less than 25 μm, as shown in Figure 6. The oxide M coatings in the present study showed oxide thickness values similar to those shown for the citric acid-based and citrus fruit-based oxide coatings in our previous studies [31,41].

### 3.3. Oxide Adhesion Quality

**Figure 7:**
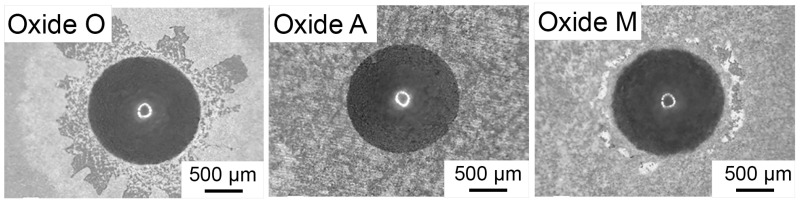
Representative VDI 3198 standard adhesion quality results for each anodized oxide group. Oxide O showed evidence of some microcracking and delamination around the indentation, indicative of unacceptable coating adhesion quality. Oxide A revealed no evidence of microcracking or delamination around the indented areas, and Oxide M showed some microcracking but no evident coating delamination. Thus, Oxide A and Oxide M were shown to exhibit acceptable coating adhesion levels according to the VDI 3198 standard testing methodology.

VDI 3198 methodology coating adhesion quality results are shown in Figure 7. Oxide O exhibited some evident coating cracking and delamination around the indentation areas. According to the VDI 3198 standard reference images, the oxide O performance represented an unacceptable qualitative coating adhesion level [49]. However, Oxide A showed no sign of microcracking or delamination around the indented areas. This finding agreed well with the XRD, SEM, oxide thickness, and EDS datasets for this oxide, which all indicated that Oxide A was a thin coating. Oxide M exhibited some microcracking around the indented areas, but no evident coating delamination. Therefore, the coating adhesion quality of Oxide A and Oxide M was considered acceptable according to the VDI 3198 standard guidelines [49]. It was interesting that oxide O, which exhibited a medium oxide thickness (Figure 6) but significantly higher Mg dopant uptake values (Figure 4A), showed extensive oxide cracking and delamination in comparison to the other oxide groups in the study.

### 3.4. Cell Viability Test Results

**Figure 8:**
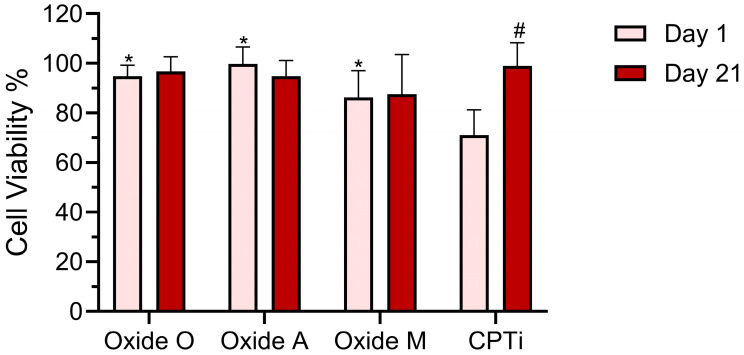
MTT assay cell viability results for each oxide and the non-anodized CPTi control group. Significant differences between the anodized groups and the non-anodized CPTi group on the same culture day are denoted with an *. Significant differences between the day 1 and day 21 results within the same test group are denoted with a #.

The day 1 and day 21 MTT assay results for each oxide and the non-anodized CPTi control group are compiled in Figure 8. A two-way repeated measures ANOVA (α = 0.05) analysis showed a significant interaction (*p* = 0.0007) between the oxide groups and the cell culture day on the MTT assay results. The main effects analyses revealed significant effects from both oxide group (*p* < 0.0001) and cell culture day (*p* = 0.0196). The post hoc Bonferroni comparisons showed each of the anodized oxide groups to exhibit significantly higher cell viabilities in comparison to the non-anodized CPTi group in the Day 1 MTT results. Specifically, the significance levels for the Oxide A, Oxide O, and Oxide M comparisons to the non-anodized CPTi group were *p* < 0.0001, *p* < 0.0001, and *p* = 0.0068, respectively. Furthermore, Oxide A showed a significantly higher day 1 cell viability compared to Oxide M, *p* = 0.0196. These findings would indicate that the anodized oxide groups showed better initial cell attachment and early cell proliferation compared to the non-anodized CPTi group. The non-anodized CPTi specimens were the only group to show a significant increase (*p* = 0.0001) in cell viability between the day 1 and day 21 study timepoints. At day 21, all anodized oxide and control groups showed statistically similar high cell viability values. Overall, the increase in early cell viability shown for each of the anodized oxide groups was attributed to the presence of the Ca-, P-, and Mg-containing oxides presenting bone-like surface chemistries.

### 3.5. Biochemical Analyses

**Figure 9:**
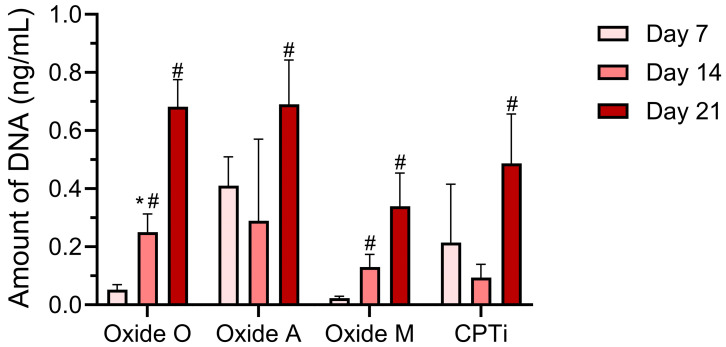
DNA assay results for each anodized oxide group and the non-anodized CPTi group on days 7, 14, and 21. Significant differences between the anodized oxide groups and the non-anodized CPTi group on the same culture day are denoted with *. Significant differences between subsequent culture day results within the same group are denoted with a #.

The day 7, 14, and 21 DNA assay results for each oxide group and non-anodized CPTi specimen group are shown in Figure 9. A two-way repeated measures ANOVA (α = 0.05) analysis showed a significant interaction (*p* < 0.0001) between the oxide groups and the cell culture day on the DNA assay results. The main effects analyses revealed significant effects from both the oxide group and the cell culture day (*p* < 0.0001). At day 7, the post hoc Bonferroni comparisons showed each oxide group to exhibit statistically similar DNA levels compared to non-anodized CPTi oxides. However, the Oxide O and Oxide A groups showed significantly higher day 7 DNA levels compared to Oxide M (*p* = 0.0037 and *p* < 0.0001). Furthermore, the Oxide O group showed significantly higher day 7 DNA levels than the Oxide A group (*p* < 0.0001). Only the Oxide O and Oxide M groups revealed significant increases in DNA between the day 7 and day 14 time points (*p* < 0.0001 and *p* = 0.0002). At day 14, Oxide O revealed statistically higher DNA levels compared to Oxide M and the non-anodized CPTi control group (*p* = 0.0019 and *p* = 0.0002). Between day 14 and day 21, each of the anodized oxide groups and the non-anodized CPTi group showed significantly increased DNA levels. Specifically, the significance levels for the increases in DNA between day 14 and day 21 for the Oxide O, Oxide A, Oxide M, and non-anodized CPTi groups were *p* < 0.0001, *p* = 0.0002, *p* = 0.0053, and *p* = 0.0006, respectively. Finally, at day 21, each of the oxide groups and non-anodized CPTi groups revealed significantly increased DNA levels compared to their day 7 levels (*p* values not included in Figure 9 for clarity). Furthermore, on day 21, the Oxide O and Oxide A DNA levels were statistically higher than those of Oxide M (*p* < 0.0001 and *p* = 0.0004). Overall, each oxide group in the present study showed DNA levels to increase over the 21-day study. Moreover, all oxide groups revealed at least statistically equivalent cellular responses compared to the non-anodized CPTi control group after 21 days. This result indicated that each of the anodized oxide groups showed good cytocompatibility.

**Figure 10:**
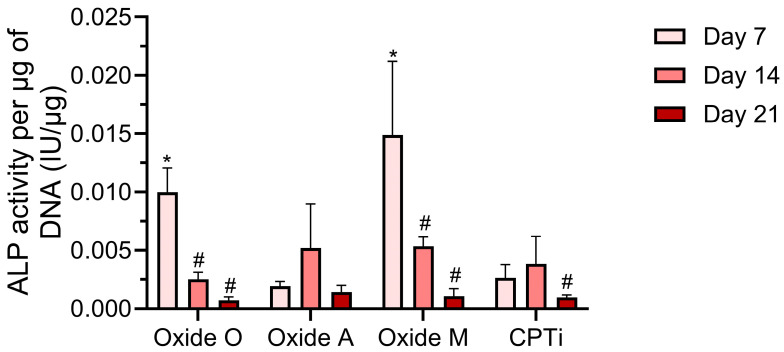
ALP assay results for each oxide group and the non-anodized CPTi group on days 7, 14, and 21. Significant differences between the anodized oxide groups and the non-anodized CPTi group on the same cell culture day are denoted with *. Significant differences between subsequent culture day results within the same group are denoted with a #.

The day 7, 14, and 21 ALP assay results for each anodized oxide group and the non-anodized CPTi group are shown in Figure 10. A two-way repeated measures ANOVA (α = 0.05) analysis showed a significant interaction (*p* < 0.0001) between the oxide groups and the cell culture day on the ALP assay results. The main effects analyses revealed significant effects from both the oxide group and the cell culture day (*p* < 0.0001). At day 7, the post hoc Bonferroni comparisons showed the Oxide O and Oxide M groups to have significantly higher ALP activity than the Oxide A (*p* < 0.0001 and *p* = 0.0016) and non-anodized CPTi (*p* < 0.0001 and *p* = 0.0021) groups. These results were attributed to the high content of Mg in Oxide O and the Ca-P-based compounds that were shown to be present in Oxide M. Oxide O and Oxide M groups also exhibited significant decreases in ALP activity between the day 7 and day 14 time points (*p* < 0.0001 and *p* = 0.0071). This result reveals that Oxides O and M attained early osteogenic differentiation and an early start to the mineralization process. In contrast, the Oxide A and non-anodized CPTi groups showed increases in ALP activity between day 7 and day 14. At day 14, the Oxide O group revealed statistically lower ALP activity compared to the Oxide M group (*p* < 0.0001 Between day 14 and day 21, the Oxide O, Oxide M, and the non-anodized CPTi groups showed a significant decrease in ALP activity (*p* = 0.0001, *p* < 0.0001, *p* = 0.0217, respectively). This result was not surprising, as it indicated the mineralization process had begun for cells growing on these groups. Oxide O showed a significantly lower ALP activity in comparison to Oxide A (*p* = 0.0339). The Oxide O and Oxide M groups revealed significantly decreased ALP activity compared to their day 7 levels (*p* values not included in Figure 10 for clarity). Overall, the Oxide O and Oxide M anodized coatings were shown to support early osteogenic differentiation and mineralization, as exhibited by the early maximum at day 7 in the ALP activity results. ALP activity serves as a marker for early osteogenic differentiation, and the ALP activity is known to decrease as the mineralization process takes place [67]. All anodized oxide groups revealed at least statistically equivalent ALP activity compared to the non-anodized CPTi control group after 21 days. These ALP results indicated that the Oxide O group, which showed high Mg dopant uptake levels via EDS, and the Oxide M group, which showed the formation of carbonated hydroxyapatite within the oxide layer according to the XRD and FTIR dataset, exhibited evidence of early osteogenic differentiation. Therefore, the Oxide O and Oxide M groups in the present study show promise to improve patient healing around titanium implants.

Two notable limitations have been identified that impact the overall interpretation of the present study findings. First, even though the Oxide M and Oxide A coatings exhibited acceptable adhesion quality, a future coating stability study over time is still warranted to understand the dissolution characteristics and the potential bone mineral ion-releasing ability of these promising coatings. Secondly, additional in vitro cell culture assessments and in vivo animal study assessments of these oxide coatings are warranted to learn more about the biological performance and overall biocompatibility of these organic acid-based oxide coatings on titanium implant alloys. We plan to explore both of these directions in future studies.

From a clinical perspective, two of the organic acid-based oxides produced in these anodization processes show promising potential for improving early-stage implant integration, as shown in the MTT and ALP assay results. Another relevant advantage of implementing these anodization processes to coat complex-shaped implants and devices is their unique ability to evenly coat complicated surface topographies. Furthermore, these organic acid-based anodization processes in this study represent cost-effective and stable implant oxide surface modifications that can easily be applied and implemented directly into implant manufacturers’ current production processes.

## 4. Conclusions

In this study, single-step anodization processes were developed to produce oxide coatings containing Ca, P, and Mg bone chemistry dopants on titanium implant surfaces to promote the osseointegration process. Each oxide surface exhibited a complex multiscale surface topography. X-ray diffraction analyses revealed combinations of anatase-phase and calcium titanate compounds. The ascorbic acid and malic acid-based oxides (Oxides A and M) also showed Ca-O formation, and the malic acid-based oxide (Oxide M) also showed tricalcium phosphate (α-TCP) and hydroxyapatite (HA) formation. Subsequent FTIR spectroscopy analyses revealed carbonate substitution peaks to be present in Oxide A and Oxide M. This finding indicated the TCP and HA compounds shown in the XRD analyses of Oxide M represented the formation of carbonated calcium phosphate compounds. The oxalic-based Oxide O group revealed the highest incorporated Mg dopant uptake levels in the study. The oxide thickness comparison showed that Oxide A was the thinnest coating and Oxide M was the thickest coating. The cell culture response to Oxide O indicated good cytocompatibility, early osteogenic differentiation, and an early start to mineralization. However, less-than-ideal adhesion quality to the titanium substrate material was shown for Oxide O, so some future refinement of the anodization parameters used in this process is warranted. In contrast, the malic acid-based Oxide M group, containing bone-like carbonated hydroxyapatite incorporation, exhibited good oxide adhesion to the titanium implant substrates. The cell culture responses to the Oxide M group also indicated good cytocompatibility and revealed early osteogenic differentiation. Overall, the Oxide O and M anodization processes coatings developed in the present study showed promise to promote osseointegration and thus improve future titanium implant clinical outcomes.

## 5. Patents

A portion of this research is part of patent application PCT/US25/18963.

## Figures and Tables

**Table 1:** Anodization process electrolytes.

Group	Distilled Water	Organic Acid Component (M)	Magnesium Phosphate (M)	Calcium Acetate (M)
**Oxide O**	500 mL	Oxalic acid—0.16	0.150	0.275
**Oxide A**	500 mL	Ascorbic acid—0.18	0.150	0.275
**Oxide M**	500 mL	Malic acid—0.1	0.100	0.350

**Table 2:** EDS-derived anodized oxide surface chemistries.

Elements	Oxide O (At. %)	Oxide A (At. %)	Oxide M (At. %)
Titanium	11 ± 0.1	10 ± 1	<1
Oxygen	64 ± 0.7	61 ± 2	58 ± 0.5
Calcium	4 ± 0.1	6 ± 0.3	18 ± 0.5
Phosphorus	7 ± 0.2	6 ± 0.2	9 ± 0.1
Magnesium	5 ± 0.2	2 ± 0.1	<1

## Data Availability

The original contributions presented in this study are included in the article. Further inquiries can be directed to the corresponding author.
